# Multimodal AI for early prediction of adverse clinical outcomes in acute pancreatitis

**DOI:** 10.1007/s00261-026-05588-w

**Published:** 2026-06-02

**Authors:** Ahmet Yasin Karkas, Yavuz B. Taktak, Burak Gultekin, Ziliang Hong, Halil Ertugrul Aktas, Deniz Seyithanoglu, Timurhan Cebeci, Alper Akin, Ece Elustu, Kerem Arisin, Ali Canturk, Mehmet Ilhan, Naci Senkal, Michael B. Wallace, Abraham F. Bezuidenhout, Frank H. Miller, Alpay Medetalibeyoglu, Mehmet Semih Cakir, Gorkem Durak, Ulas Bagci, Sukru Mehmet Erturk

**Affiliations:** 1 Department of Radiology, Istanbul University Faculty of Medicine, Istanbul, Turkey; 2 Department of Internal Medicine, Uskudar State Hospital, Istanbul, Turkey; 3 Department of Radiology, Northwestern University, Chicago, United States; 4 Department of Internal Medicine, Istanbul University Faculty of Medicine, Istanbul, Turkey; 5 Department of Public Health, Marmara University School of Medicine, Istanbul, Turkey; 6 Department of Radiology, Ministry of Health University Abdulhamid Han Training and Research Hospital, Istanbul, Turkey; 7 Department of General Surgery, Istanbul University Faculty of Medicine, Istanbul, Turkey; 8 Department of Medicine, Mayo Clinic, Jacksonville, United States; 9 Department of Radiology, Beth Israel Deaconess Medical Center, Boston, United States

**Keywords:** Acute pancreatitis, Artificial intelligence, Deep learning, Radiomics, Multimodal AI, Contrast-enhanced computed tomography, Explainable artificial intelligence, Outcome prediction

## Abstract

**Background:**

Conventional clinical scoring systems and contrast-enhanced computed tomography (CECT) interpretation provide limited accuracy in predicting adverse outcomes in early acute pancreatitis (AP). This leads to suboptimal patient management and underscores the need for improved triage methods. To address this, we developed a multimodal artificial intelligence (AI) framework that integrates clinical parameters, radiomics, and deep learning (DL) models to predict adverse clinical outcomes in early AP.

**Methods:**

In this retrospective tertiary-care, imaging-enriched cohort study, patients with AP who underwent CECT within 72 h of hospital admission were included. Adverse clinical outcomes were defined as mortality, intensive care unit (ICU) admission, or the need for invasive intervention within 30 days. Radiomics (using both pancreatic and peripancreatic features) and DL models were developed using CECT images to predict adverse outcomes. Multimodal models were constructed by integrating imaging and laboratory variables. Model performance was compared with three independent radiologists’ prognostic imaging assessments and with established clinical scoring systems (Ranson and Glasgow-Imrie).

**Results:**

A total of 284 patients with AP were included, of whom 140 (49.3%) experienced adverse clinical outcomes. Conventional clinical scores showed limited discrimination, with AUCs of 0.61 for Ranson and 0.67 for Glasgow-Imrie. Imaging-only assessment by three expert radiologists yielded modest predictive performance (average AUC = 0.629; sensitivity = 42.5%, specificity = 83.2%) and moderate interobserver agreement (Fleiss κ = 0.650; ICC = 0.653). Imaging-only radiomics and DL models achieved higher discrimination (AUC 0.77 and 0.76, respectively). Integration of laboratory parameters into the radiomics model further improved predictive performance (AUC of 0.77 to 0.80), whereas the DL and fusion models showed no substantial improvement.

**Conclusion:**

Our multimodal AI framework, which combines quantitative CECT features with clinical data, enhances the ability to predict adverse outcomes in early AP compared to traditional clinical and imaging severity scoring systems. These findings should be interpreted as preliminary, and prospective multicenter validation is required before considering clinical implementation.

**Supplementary Information:**

The online version contains supplementary material available at https://doi.org/10.1007/s00261-026-05588-w.

## Introduction

Acute pancreatitis (AP) is a frequent inflammatory condition of the pancreas, characterized by its acute onset and rapid progression [1]. AP represents one of the most common causes of hospitalization for gastrointestinal diseases, with a reported global incidence of approximately 30–40 cases per 100,000 population annually [2]. Additionally, the economic impact of AP is substantial, accounting for more than 255,000 hospital admissions annually in the United States and an estimated aggregate inpatient cost of approximately USD 3 billion per year, with total hospital charges exceeding USD 12 billion [3–5]. Although most cases are mild and self-limiting, up to 30% of patients are at risk of progressing to more severe stages, characterized by persistent organ failure and local complications such as infected pancreatic necrosis [6, 7]. This progression significantly increases morbidity (40–60%) and mortality (15–30%), underscoring the importance of early recognition and timely intervention [2].

Timely identification of high-risk patients enables prompt supportive management, which reduces complications and organ failure [8, 9]. To address this need, several clinical scoring systems, such as the Ranson and Glasgow-Imrie scores, have been developed to stratify disease severity [10, 11]. However, these systems have suboptimal performance and rely on serial measurements obtained within the first 48 h, which limits their value for early risk assessment [12, 13]. This delay may hinder recognition of patients who initially appear clinically stable but later progress to persistent organ failure or require intensive care. Moreover, imaging-based severity assessment -Balthazar computed tomography severity index (CTSI) [14]- does not align well with clinical courses. Patients with extensive pancreatic and peripancreatic changes may have a favorable course, whereas those with relatively mild imaging findings can develop persistent organ failure and adverse outcomes [15, 16]. This imperfect alignment demonstrates the limitations of relying on rule-based methods focused on a single modality, thus justifying the need for more adaptable, data-driven, and comprehensive approaches.

Advanced imaging analysis has moved beyond purely visual interpretation. These methods extract quantitative information from images and reveal clinically meaningful patterns related to underlying tissue characteristics [17]. Radiomics was one of the first structured approaches to capture features such as intensity, texture, and spatial arrangement [18]. It has been widely applied in oncologic and abdominal imaging for tumor characterization, differentiation, staging, and prediction of clinically relevant outcomes, including lymph node metastasis and disease progression, reflecting its increasing clinical relevance in quantitative imaging [19–21]. Deep learning (DL), in turn, learns complex image patterns directly from the data without predefined feature engineering [22]. Both methods are now widely used in medical imaging for diagnosis, risk stratification, prognosis, and prediction of treatment response [23–25]. Together, they have expanded the role of imaging from visual assessment to quantitative modeling.

These advances have extended to AP, with previous studies showing that AI models based on imaging features can outperform traditional severity scoring systems [26, 27]. However, most current approaches rely on a single data modality, whereas real-world clinical decision-making integrates imaging, laboratory, and clinical data. Although multimodal AI methods have been proposed, evidence comparing their benefits to those of single-modality models remains limited [28, 29]. It remains uncertain whether imaging-derived signals reflect only systemic inflammation or also detect early disease processes that independently cause worse outcomes. Additionally, combining diverse high-dimensional data, such as clinical, laboratory, and imaging data, into a single model increases complexity. This can also obscure the influence of individual variables, making models harder to interpret and potentially limiting clinical usefulness. In this context, explainable artificial intelligence (XAI) provides a clear way to assess how each feature contributes, enhancing transparency and supporting biologically meaningful improvements in the model [30].

The primary objectives of this research are:

1. Model Development and Evaluation: To develop and assess radiomics, DL, and hybrid fusion models designed for the early, robust, and noninvasive prediction of outcomes in AP patients.
2. Multimodal Feature Integration: To determine whether incorporating imaging features with clinical data improves AP outcome prediction beyond the performance of imaging-only models.
3. Performance Comparison with Experts: To benchmark the predictive accuracy of the AI models against expert radiologists’ visual severity assessments.
4. Increasing Model Interpretability: Application of advanced XAI techniques to facilitate clinical comprehension and adoption.

## Methods

### Data collection

Ethical approval was obtained from the institutional review board, with a waiver of informed consent due to the study’s retrospective nature.

All patients who were admitted to our center with AP between January 2012 and December 2025 were screened for inclusion. Eligibility required a contrast-enhanced computed tomography (CECT) performed within 72 h of admission. In addition, complete clinical records were required for inclusion to ensure accurate calculation of the Ranson and Glasgow-Imrie scores and reliable ascertainment of 30-day clinical outcomes. This included full documentation of admission laboratory parameters -white blood cell count (WBC), hematocrit, serum glucose, blood urea nitrogen (BUN), creatinine (Cr), C-reactive protein (CRP), lactate dehydrogenase (LDH), albumin, and calcium - as well as 48-h follow-up laboratory values. Vital signs and hemodynamic parameters (including blood pressure and vasopressor requirement) were also recorded, along with detailed in-hospital outcomes, including intensive care unit (ICU) admission, need for invasive intervention, and mortality. Patients with missing laboratory measurements or incomplete outcome documentation were excluded to maintain methodological consistency across comparative analyses.

Adverse clinical outcomes were defined as the occurrence of at least one of the following within 30 days of symptom onset:

1. Admission to an ICU, and/or.
2. The need for an intervention, and/or.
3. All-cause mortality.

Interventions included endoscopic retrograde cholangiopancreatography (ERCP), percutaneous catheter drainage, endoscopic or surgical necrosectomy, biliary stenting, pancreatic duct stenting, and open surgical procedures such as laparotomy. These categories were selected in accordance with current clinical guidelines and the literature on invasive management strategies for AP [31, 32].

**Fig. 1:**
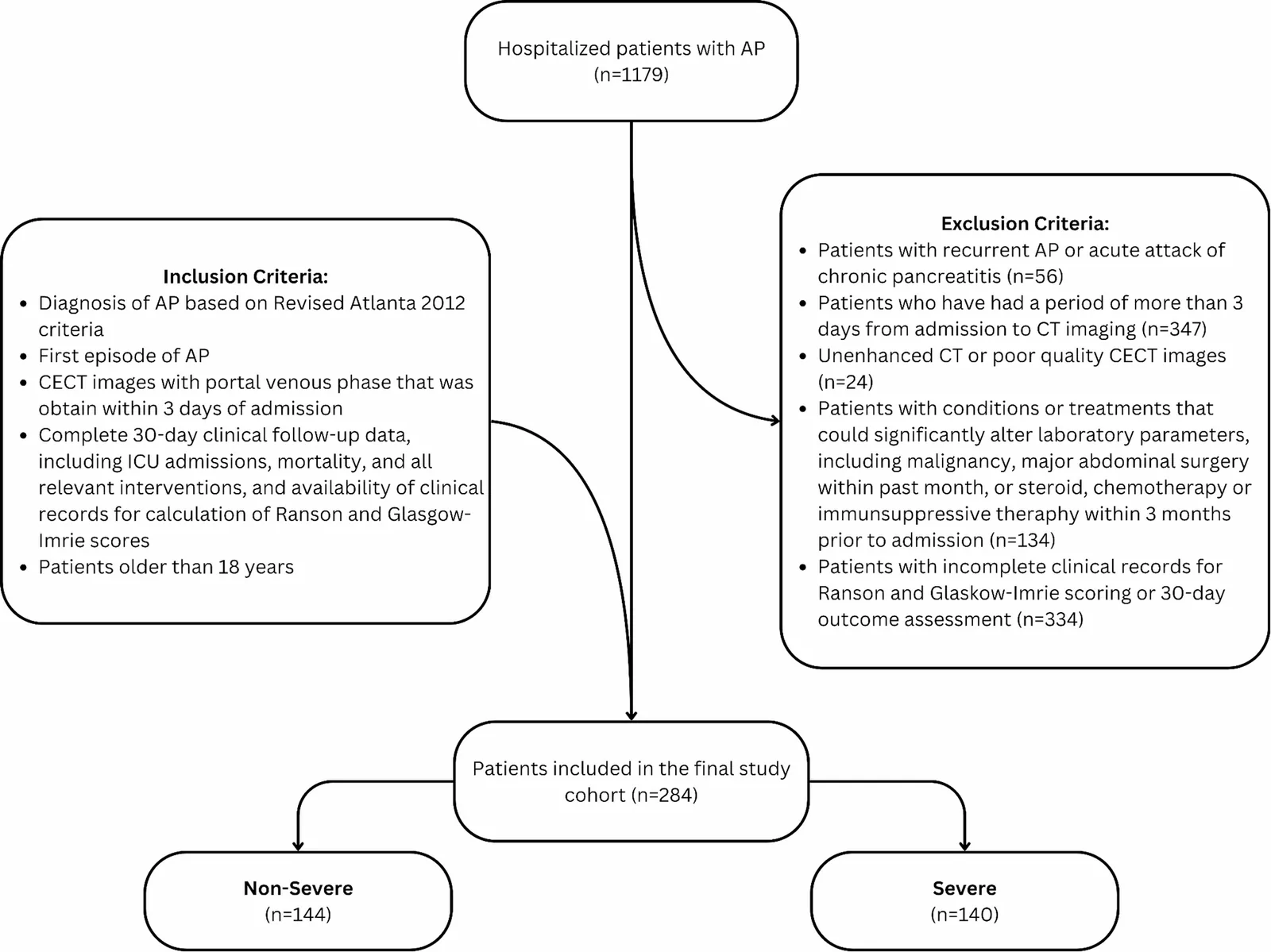
Flowchart for patient selection

The flow chart for patient selection and evaluation is summarized in Fig. 1.

### CT image acquisition and image interpretation

**Fig. 2:**
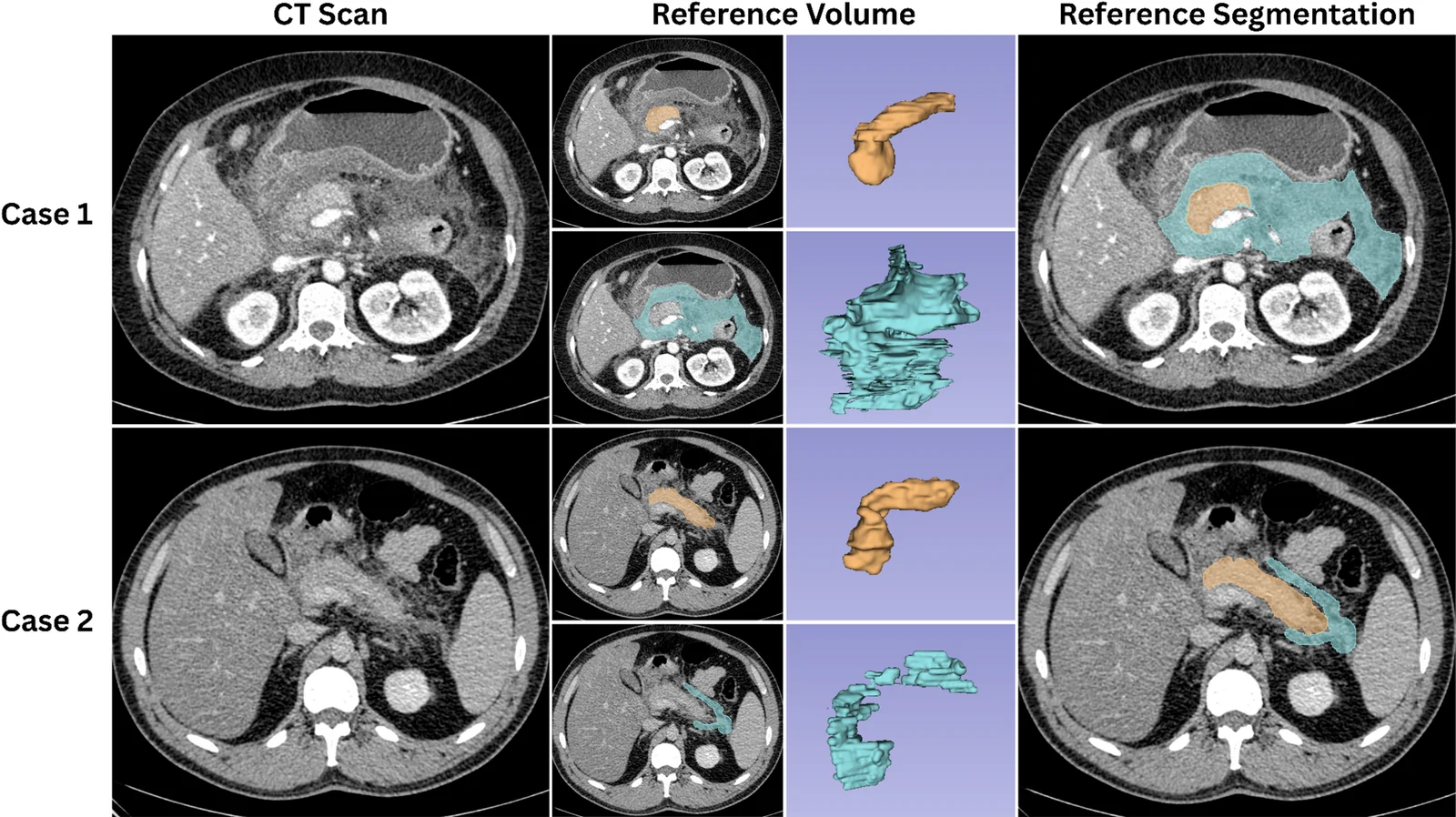
Segmentation of the pancreas (yellow color) and peripancreatic (green color) region of cases clinically diagnosed as mild (**case 1**) and severe (**case 2**) AP in LIFEx software

CECT scans were performed using Canon TSX-305 A/5K and Brilliance-Philips scanners; additional acquisition details are provided in the Supplementary Material. The images were retrospectively collected from the Picture Archiving and Communication System (PACS). Two abdominal radiologists, each with five years of experience, manually performed volumetric three-dimensional (3D) segmentation of the pancreas and peripancreatic edema using Local Image Features Extraction (LIFEx) 7.4.3 software [43], remaining blinded to each other. A senior abdominal radiologist with 15 years of experience confirmed the segmented volumes for quality and consistency. Figure 2 shows the segmentation of the pancreas and peripancreatic region in patients with AP of varying severity.

### Intra-observer reliability and inter-observer agreement for segmentation

We calculated intra- and inter-observer agreements using the Dice Similarity Coefficient (DSC) and Cohen’s kappa scores. To assess inter-observer reliability, a random sample of 30 CT scans was used. The inter-observer agreement yielded DSCs of 82.9% and 74.8% for the pancreas and peripancreatic regions, respectively. To assess intra-observer agreement, the same radiologist re-evaluated 20 randomly selected CECT scans after a three-week wash-out period. Intra-observer agreement was high, with DSCs of 87.1% and 80.5% for the pancreas and peripancreatic regions, respectively. Cohen’s kappa coefficients exceeded 0.70 for both the pancreas and peripancreatic areas of both analyses, indicating good consistency.

### Radiologist-based imaging-only assessment for outcome analysis

Three independent radiologists with 5 to 7 years of experience reviewed the CECT scans in a blinded manner, using only the imaging data. They were informed of the adverse clinical endpoints and asked to provide a binary prognostic assessment for each case. Their evaluation was based on a comprehensive visual interpretation of pancreatic and peripancreatic findings, including the extent of peripancreatic inflammation, the presence of pancreatic necrosis, and additional extrapancreatic features on abdominal CECT that could influence clinical outcomes consistent with the principles underlying the Balthazar CT Severity Index [14]. Inter-reader agreement was assessed using Cohen’s kappa for pairwise comparisons and Fleiss’ kappa for overall agreement. A majority-vote consensus was then compared with clinical outcomes using ROC analysis.

### Radiomics analysis

All CECT images were resampled to an isotropic voxel spacing of 1.0 × 1.0 × 1.0 mm³ and intensity-normalized to reduce inter-subject variability. Radiomics features were extracted from both the pancreas and peripancreatic edema regions using PyRadiomics (version 3.0.1), including first-order statistics, shape-based descriptors, and texture matrices. Wavelet-transformed and Laplacian-of-Gaussian filtered features were additionally computed to capture multi-scale textural information, yielding 1409 features per region (2818 total). Given the high feature-to-sample ratio, a two-stage feature selection strategy was implemented to reduce dimensionality, enhance feature stability, and limit overfitting. This process included variance filtering, outcome correlation analysis, inter-feature correlation removal, stability-based selection via repeated cross-validation (1000 iterations), and L1-regularized logistic regression. To capture nonlinear relationships, polynomial feature expansion (degree = 2) was applied, with all selection and expansion steps performed independently within each training fold to prevent data leakage. The selected radiomic features were further categorized according to their quantitative imaging characteristics and potential biological relevance in acute pancreatitis, as summarized in Supplementary Table S1. Additionally, routinely available laboratory variables - including WBC, BUN/creatinine ratio, CRP, LDH, and albumin- were incorporated alongside imaging features.

### Deep learning analysis

A smoothed pancreas mask was applied to the CECT volumes, and the masked region was cropped and resized to 96 × 96 × 128 voxels based on median pancreas dimensions. A three-dimensional DenseNet-264 architecture was trained from scratch using the Adam optimizer (learning rate 1 × 10⁻³, decay factor 0.5 every 30 epochs), a batch size of 8, cross-entropy loss, and 300 training epochs, with random spatial rotations for augmentation [33]. To restrict feature aggregation to pancreatic tissue, masked global average pooling was applied by resizing the pancreas mask to the feature map resolution and pooling only within the masked region. The pooled representation was passed through a fully connected layer to generate class logits, which were subsequently combined with the same laboratory variables.

### Fusion model development

**Fig. 3:**
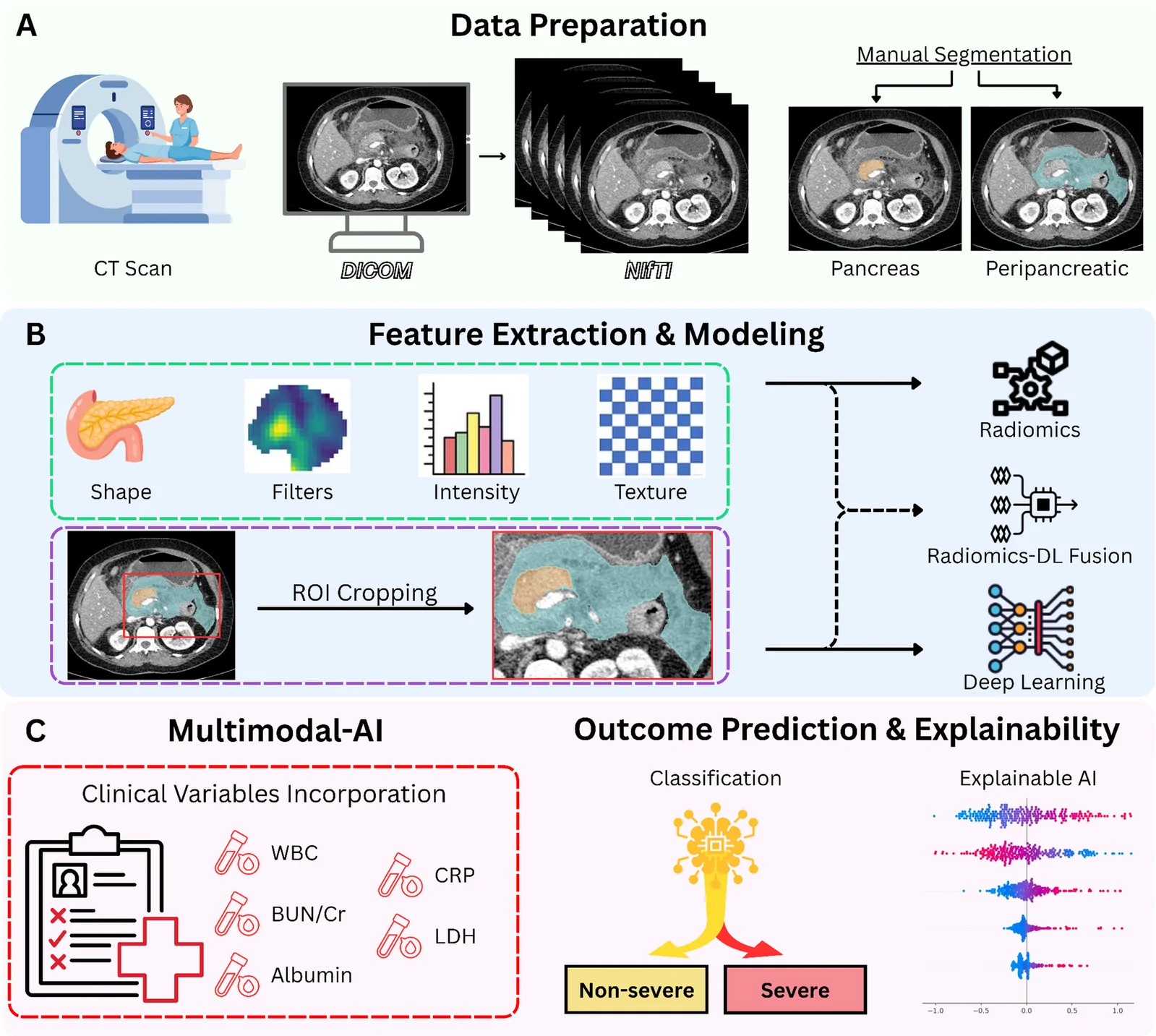
Representative pipeline overview of our methodology

A stacking-based fusion framework integrated DL logits, radiomics decision scores, and laboratory variables into a multimodal feature vector. Two base models—DenseNet-264 and L1-regularized logistic regression—were trained independently, and a logistic regression meta-learner was trained on their concatenated outputs within each fold of a stratified five-fold cross-validation scheme to prevent data leakage. Performance was evaluated across logistic regression, support vector machines, random forests, and XGBoost [34]. SHapley Additive exPlanations (SHAP) analysis was performed to enhance model interpretability by identifying the most influential features driving model predictions (Fig. S1) [35]. Figure 3 shows the representative pipeline overview of our methodology.

### Statistical analysis

Continuous variables were reported as median (Interquartile Range [IQR]), while categorical variables were expressed as numbers and percentages [n (%)]. The normality of continuous variables was assessed using the Kolmogorov–Smirnov and Shapiro–Wilk tests. The Mann–Whitney U test was used to compare continuous variables between two independent groups, whereas the chi-square test was used to compare categorical variables between groups. A p-value < 0.05 was considered statistically significant. We compared each ML model with the best-performing traditional scoring method using a paired t-test. DeLong’s test was used to compare the AUCs of the radiomics-only and multimodal models on the same held-out test set. Bootstrap resampling with 1000 iterations at the patient level was used to estimate 95% confidence intervals. All statistical analyses were conducted using IBM SPSS Statistics for Windows, version 29 (IBM Corp., Armonk, NY, USA).

## Results

A total of 284 patients with AP were included in the analysis. The cohort consisted of 153 (53.8%) men and 131 (46.2%) women, with a mean age of 53.1 ± 16.1 years. The median Ranson score was 2.0 (IQR 2.0), and the median Glasgow-Imrie score was 1.0 (IQR 2.0). Biliary pancreatitis was the most common etiology (46.1%), followed by other causes (37.3%), hyperlipidemia (14.8%), and alcohol-related pancreatitis (1.8%). Nearly half of the patients were classified as severe (49.3%), while 50.7% were non-severe, as shown in Table 1. Severe disease was defined as the occurrence of at least one adverse outcome, including admission to the ICU (*n* = 50), all-cause mortality (*n* = 21), or the need for intervention for AP or its complications (*n* = 71). Among the interventions, 42 were ERCP procedures, 19 were drainage procedures, and 7 were necrosectomies; the remaining interventions were performed for other AP-related indications (cystogastrostomy, tube cholangiostomy, endoscopic ultrasound, etc.).

**Table 1:** Baseline characteristics of patients with AP categorized by severity

Characteristics	Group	Non-Severe (*N* = 144)	Severe (*N* = 140)		*P*
Sex n (%)	Male	70(45.7)	83(54.2)		0.098*
	Female	74(56.5)	57(43.5)		
Etiology n (%)	Biliary	44(33.6)	87(66.4)		< 0.001*
	Hyperlipidemia	26(61.9)	16(38.1)		
	Alcoholic	2(40.0)	3(60.0)		
	Others	72(67.9)	34(32.1)		
Age mean ***±*** SD		49.4 *±* 16.2	56.9 *±* 15.1		< 0.001***
Ranson median (IQR)		1.00(2.00)	2.00(3.00)		< 0.001**
Glasgow-Imrie median (IQR)		1.00(1.00)	2.00(2.00)		< 0.001**
WBC median (IQR)		10450.00 (5870.00)	12750.00(8150.00)		< 0.001**
Hematocrit median (IQR)		39.42(7.48)	39.75(7.83)		0.867**
Glucose median (IQR)		124.00(45.50)	134.00(77.00)		0.028**
BUN median (IQR)		12.00(8.63)	14.00(10.08)		0.006**
Creatinine median (IQR)		0.80(0.30)	0.83(0.30)		0.098**
BUN/Cr median (IQR)		15.74(8.41)	17.73(8.95)		0.037**
CRP median (IQR)		12.79(53.81)	46.00(138.00)		< 0.001**
LDH median (IQR)		262.00(151.00)	328.00(221.00)		0.001**
Albumin median(IQR)		4.28(0.61)	3.99(1.05)		< 0.001**
Calcium median(IQR)		9.20(0.62)	9.07(0.90)		0.006**

Continuous variables are presented as Mean ± SD or Median (IQR), while categorical variables are presented as n (%), SD(Standard Deviation), and IQR(Interquartile Range)

*Chi Square Test ** Mann Whitney U Test *** Independent Sample T Test

The Ranson and Glasgow–Imrie scores showed limited discriminative performance, with AUC values of 0.61 and 0.67, respectively. Sensitivity remained moderate (49.3% for Ranson and 59.2% for Glasgow–Imrie), while specificity ranged from 72.8% to 74.8%. Detailed performance metrics of both scoring systems are summarized in Table 2. Radiologist-based assessment also demonstrated limited predictive performance for adverse AP outcomes, with a mean AUC of approximately 0.63 and a low average sensitivity of 42.5% (Table 2). Majority voting yielded an AUC of 0.61. Interobserver agreement among the three radiologists was moderate (Fleiss κ = 0.650; ICC = 0.653).

**Table 2:** Performance of the Ranson and Glasgow-Imrie scores and radiologist visual assessment

	AUC/Balanced Accuracy (%)	Accuracy (%)	Precision (PPV) (%)	Recall (Sens) (%)	Spec (%)	F1-Score (%)	NPV (%)
Ranson*	61.0	61.2	63.6	49.3	72.8	55.6	59.8
Glasgow-Imrie**	67.0	67.1	69.4	59.2	74.8	64	65.5
Radiologist-1	64.4	64.7	70.0	49.3	79.6	57.9	61.9
Radiologist-2	62.2	62.6	75.8	35.2	89.1	48.1	58.7
Radiologist-3	62.0	62.3	68.5	43	81.0	52.8	59.5
Average	62.9	63.2	71.4	42.5	83.2	52.9	60.0
Majority Vote	60.9	60.9	68.8	38.7	83.0	49.5	58.4

*Ranson ≥ 3

**Glasgow ≥ 2

Without incorporating laboratory parameters, all three models achieved comparable discriminatory performance. The radiomics model demonstrated the highest overall balance across metrics, with an AUC of 76.99 (95% CI: 0.71–0.82), accuracy of 71.29 ± 4.56%, and NPV of 71.90 ± 3.70%. The DL model yielded a similar AUC (76.88 ± 6.78%) while achieving the highest PPV (72.45 ± 7.56%) and specificity (80.34 ± 12.85%) among the three models. The fusion model did not outperform the individual models, producing an AUC of 76.56 ± 6.50% and an accuracy of 70.25 ± 5.00%. All detailed results, including complete metrics, are shown in Table 3.

**Fig. 4:**
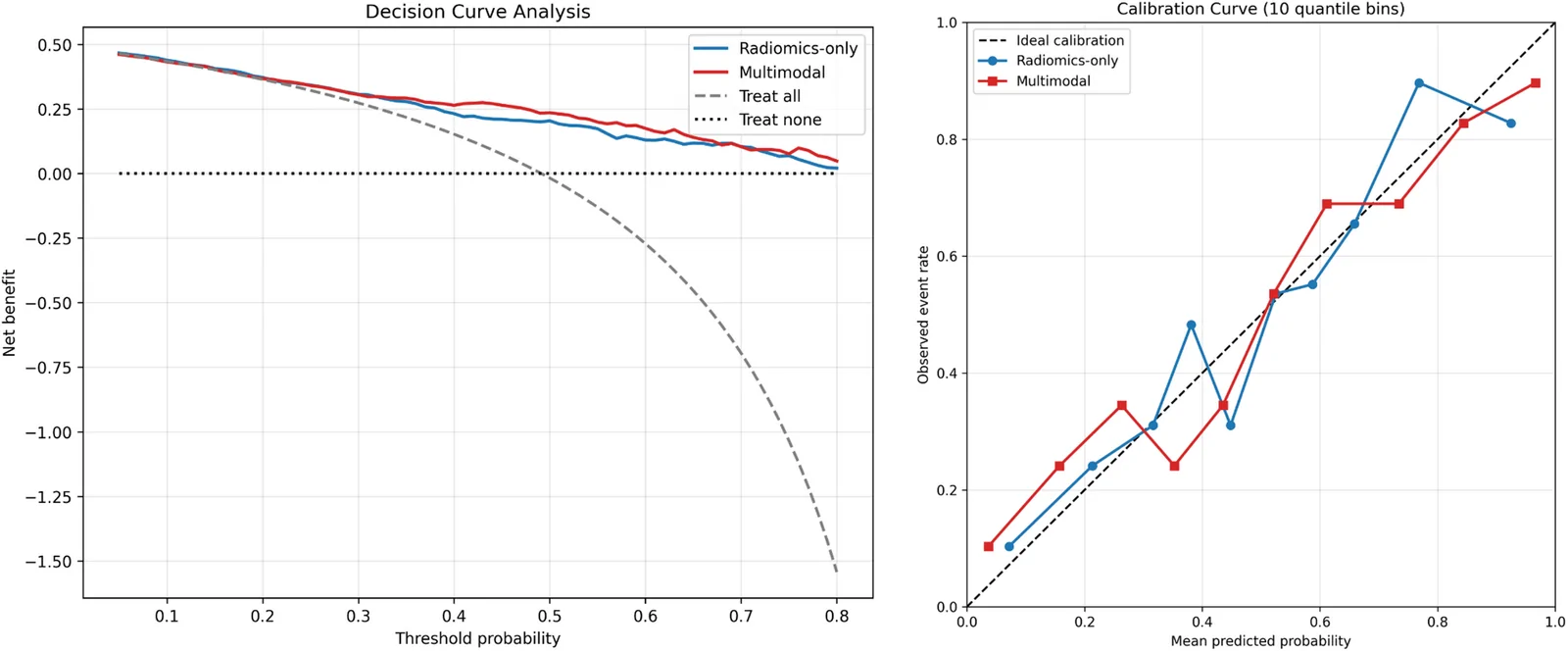
Decision and calibration curve analysis of model performance. Left panel: Decision curve analysis showing net benefit across a range of threshold probabilities for the radiomics-only (blue line) and multimodal model (red line), along with treat-all and treat-none strategies. Right panel: Calibration curves showing the relationship between mean predicted probabilities and observed event rates for the radiomics-only and multimodal models, with the ideal calibration line shown for reference

Incorporation of laboratory parameters yielded the most notable performance improvement in the radiomics model, which achieved the highest results across nearly all metrics: AUC of 79.83 (+2.84%; 95% CI: 0.74–0.85; *p* = 0.03), accuracy of 74.40 ± 3.66% (+3.11%), sensitivity of 74.41 ± 3.68% (+3.01%), and specificity of 76.94 ± 7.50% (+4.69%) compared to the model without laboratory parameters. The DL model showed a marginal AUC improvement (76.95 ± 6.88%) and retained the highest specificity among the three models (78.25 ± 3.69%), though its overall performance remained below that of the radiomics model. The fusion model again did not surpass the other models, with an AUC of 76.63 ± 6.57% and an accuracy of 70.60 ± 6.04%. All detailed results, including complete metrics, are shown in Table 4. Decision curve and calibration analyses showed comparable clinical net benefit and generally acceptable calibration for both the radiomics-only and multimodal models (Fig. 4).

**Table 3:** Performance of the radiomics, DL, and fusion models without incorporating laboratory parameters

Model	AUC (%)	Accuracy (%)	Precision (PPV) (%)	Recall (Sens) (%)	Spec (%)	F1-Score (%)	NPV (%)
Radiomics	**76.99 ± 4.35**	**71.29 ± 4.56**	72.09 ± 4.70	**71.40 ± 4.42**	72.25 ± 13.00	**71.09 ± 4.58**	**71.90 ± 3.70**
DL	76.88 ± 6.78	70.60 ± 7.24	**72.45 ± 7.56**	70.44 ± 7.58	**80.34 ± 12.85**	69.62 ± 7.53	68.89 ± 7.39
Fusion	76.56 ± 6.50	70.25 ± 5.00	70.45 ± 4.93	70.11 ± 5.18	76.21 ± 2.14	70.01 ± 5.30	68.99 ± 4.87

Bold values indicate the best-performing model for each performance metric

*Data are presented as mean ± standard deviation

**Table 4:** Performance of the radiomics, DL, and fusion models incorporating five laboratory parameters (WBC, BUN/creatinine, CRP, LDH, and albumin)

Model	AUC (%)	Accuracy (%)	Precision (PPV) (%)	Recall (Sens) (%)	Spec (%)	F1-Score (%)	NPV (%)
Radiomics	**79.83 ± 4.75**	**74.40 ± 3.66**	**74.71 ± 4.10**	**74.41 ± 3.68**	76.94 ± 7.50	**74.34 ± 3.61**	**73.96 ± 3.18**
DL	76.95 ± 6.88	71.29 ± 6.59	71.58 ± 6.54	71.12 ± 6.79	**78.25 ± 3.69**	70.97 ± 6.98	69.65 ± 6.17
Fusion	76.63 ± 6.57	70.60 ± 6.04	70.78 ± 6.04	70.46 ± 6.18	76.21 ± 3.96	70.38 ± 6.27	69.41 ± 5.70

Bold values indicate the best-performing model for each performance metric

*Data are presented as mean ± standard deviation

## Discussion

In this retrospective, single-center cohort of 284 patients, we developed a spectrum of CECT-based AI predictive models, encompassing radiomics, DL, fusion, and multimodal data integration, to predict adverse clinical outcomes in early AP. Our findings demonstrate that conventional clinical scoring systems showed limited predictive performance, with AUC values of 0.61 for the Ranson score and 0.67 for the Glasgow-Imrie score. Radiologists’ visual assessment demonstrated similarly modest accuracy (average AUC ≈ 0.63), low sensitivity (42.5%), high specificity (83.2%), and only moderate interobserver agreement (Fleiss κ = 0.650; ICC = 0.653). The high specificity but low sensitivity indicates that radiologists mainly identified high-risk cases when early CECT findings were obvious. Conversely, some patients who later experienced adverse outcomes only showed subtle abnormalities on imaging, which explains the limited sensitivity of visual evaluation. In AI models, image-only radiomic and DL models achieved higher AUC values of 0.77 and 0.76, respectively. Combining radiomic and DL features did not enhance performance, indicating that the two methods capture overlapping information in early AP.

Performance differed across modeling strategies when integrating multimodal data. The radiomics-clinical model achieved the highest discrimination (AUC = 0.80), whereas the DL-laboratory model and the combined radiomics-DL-laboratory fusion model remained at an AUC of 0.76. Including clinical parameters in the radiomics-based multimodal model led to the greatest improvement compared to other multimodal setups. This indicates that radiomic features may provide a more biologically relevant view of early structural changes in AP, enabling better integration with systemic biomarkers. In our cohort, SHAP analysis demonstrated notable contributions from original_shape_MeshVolume, original_glrlm_RunLengthNonUniformity, and wavelet-LLL_firstorder_Skewness features extracted from the pancreatic region, as well as lbp-2D_firstorder_Mean and wavelet-HLH_glszm_GrayLevelNonUniformity features extracted from the peripancreatic edema region. These features may reflect inflammatory tissue heterogeneity, edema complexity, morphologic pancreatic alteration, and evolving necrotic change in acute pancreatitis. Ultimately, these findings demonstrate that cross-domain complementarity in multimodal risk estimation can be achieved using parameters routinely collected in clinical practice, without significantly increasing the model’s complexity.

In our cohort, 49.3% of patients were classified as severe, whereas 50.7% were categorized as non-severe. The relatively high proportion of severe cases compared to the literature should be interpreted in light of the composite endpoint selected. Although this distribution may initially raise concerns about potential selection bias, it more plausibly reflects the imaging-based inclusion criteria and the tertiary referral setting, in which early CECT is more frequently performed in patients with more advanced pancreatitis, whereas mild pancreatitis is managed without imaging. Importantly, all consecutive eligible patients meeting predefined imaging and data availability criteria were included, reducing the likelihood of arbitrary case selection. Thus, the observed severity distribution likely represents enrichment inherent to study design rather than systematic selection bias. In addition, ICU admission and mortality represent unequivocal markers of clinical deterioration, while invasive interventions were included to capture clinically meaningful escalation of care beyond supportive management. ERCP was considered an adverse outcome only when performed for AP-related cholangitis or ongoing biliary obstruction, representing guideline-supported therapeutic intervention rather than routine diagnostic ERCP. In contrast, drainage, necrosectomy, and related procedures more directly reflected the need for procedural management of complicated AP [13, 31]. Therefore, this endpoint should be viewed as capturing clinically meaningful adverse progression rather than a single homogeneous severity category.

The selection of clinical parameters is biologically grounded and supported by prior evidence in AP. BUN reflects intravascular volume depletion and renal hypoperfusion secondary to third-spacing and systemic inflammation [36]. LDH, incorporated in the original Ranson criteria [10], serves as a marker of cellular injury and tissue necrosis and has been associated with more severe disease courses and organ dysfunction. CRP is a well-established inflammatory marker, and levels above 150 mg/L within 48 h are linked to severe AP and pancreatic necrosis [37]. Hypoalbuminemia has been associated with persistent organ failure and mortality [38], while elevated WBC reflects early systemic inflammatory activation and progression to organ dysfunction [39]. Together, these markers capture key biological aspects of early AP and complement the imaging features in the multimodal model.

In our study, conventional clinical scoring systems had a limited ability to predict adverse outcomes in the early phase of AP, which aligns with the current evidence. The Ranson and Glasgow-Imrie scores rely on predefined variables and laboratory measurements obtained at 48 h, thereby limiting their usefulness for early risk stratification and critical management decisions, such as intensive monitoring or ICU admission. Moreover, prognostic assessment based on static laboratory parameters may not adequately reflect the dynamic and rapidly evolving course of AP [6, 13, 32]. In our previous study, radiomic features derived from CECT demonstrated superior discriminative performance compared with Ranson and Glasgow-Imrie scores for predicting AP severity (AUC = 0.77 vs. 0.59–0.69) [40]. These findings highlight that conventional clinical scores rely on predefined and delayed laboratory variables, whereas DL-based imaging captures CECT patterns that reflect early inflammatory and tissue changes not detected by routine clinical parameters.

Previous studies have explored multimodal AI approaches for risk stratification in AP by integrating imaging-derived features with clinical data. As parallel with our study, Yin et al. developed a multimodal model that combines clinical variables with CECT-based radiomics and DL features to predict severe AP, demonstrating that integrating multiple information sources yields a clearer and more accurate assessment of disease severity than relying on either clinical or imaging features alone [28]. Their fusion model (PrismSAP) achieved excellent discrimination, with an external validation AUC of 0.916, clearly outperforming traditional scoring systems such as Ranson and BISAP. Their work demonstrated the value of integrating multiple data types for risk stratification. By contrast, our study focuses on predicting clinically meaningful adverse outcomes, including mortality, ICU admission, and the need for invasive interventions, rather than on severity classification alone. These findings align with growing evidence that integrated AI frameworks are well-suited to capturing the complexity of AP and enabling more reliable early risk stratification.

Similarly, Qi et al. developed a combined clinical-radiomics model that incorporated CT radiomic features with laboratory parameters, including serum calcium, CRP, and WBC, achieving AUC values above 0.90 and demonstrating superior net clinical benefit compared with single-modality models [29]. Their work focused on severity stratification using the Revised Atlanta classification and structured clinical and imaging variables. In contrast, our study targets clinically meaningful adverse outcomes, such as mentioned before. Moreover, whereas Qi et al. primarily compared their model with clinical-only and radiomics-only approaches, we further benchmarked imaging-based AI performance against radiologist assessments and established clinical scoring systems, thereby extending prior clinical–radiomics frameworks toward outcome-oriented, clinically actionable prediction. Consistent with established clinical understanding of acute pancreatitis, our SHAP analysis showed that higher WBC, BUN/Cr, LDH, and CRP values, as well as lower albumin levels (hypoalbuminemia), were associated with an increased probability of a poor prognosis.

Radiologists’ assessment demonstrated moderate interobserver agreement (Fleiss κ = 0.650; ICC = 0.653) and limited predictive performance (average AUC = 0.629). This reflects a well-recognized limitation of AP: previously defined radiologic findings do not consistently correlate with subsequent clinical deterioration. Adverse clinical outcomes are driven by complex inflammatory and systemic processes that may not be visible to the human eye [6, 15, 16]. In addition, early CECT findings frequently lack clear categorical boundaries, necessitate subjective interpretation, and thereby increase interobserver variability. Consequently, qualitative CECT interpretation alone is insufficient for reliable early prognostication.

Our study has several limitations. First, the absence of an external, independent hold-out validation cohort is the principal limitation of this study. Although model development and evaluation were performed using internal cross-validation, this design may still yield optimistic performance estimates and limit broader generalizability. Therefore, the findings should be regarded as preliminary and require validation in independent, prospective, multicenter cohorts before clinical implementation. Second, although the Revised Atlanta classification [6] distinguishes transients from persistent organ failure, we focused on sustained organ failure to define clinically meaningful outcomes and enable robust model training. In addition, the composite outcome used in this study combined ICU admission, intervention, and mortality, which differ in clinical severity and decision-making context. Although these events were grouped to capture clinically meaningful adverse progression relevant to early risk stratification, this approach introduces heterogeneity that should be considered when interpreting the results. While this approach facilitated a balanced distribution across groups, it may not fully reflect real-world severity patterns, in which most cases are mild. Third, inclusion was restricted to patients undergoing early CECT, which may limit applicability to centers that do not routinely perform early imaging. Fourth, relying on manual 3D segmentation by radiologists limits reproducibility and scalability because it introduces potential operator variability, even with expert oversight and reproducibility checks. However, the literature includes some advances in automating this pipeline [41, 42]. Fifth, laboratory parameters were analyzed at a single time point, potentially overlooking dynamic changes relevant to prognosis. Finally, radiologist assessment was performed without clinical information to ensure methodological comparability, thereby not fully replicating real-world practice. Despite these limitations, consistent findings across imaging, clinical, and reader-based comparisons support the potential role of multimodal quantitative analysis for early risk stratification in AP.

## Conclusion

This study demonstrated that a multimodal framework integrating CECT-based radiomics and DL models with clinical data enables early and accurate prediction of adverse outcomes in AP. This approach leverages imaging features alongside markers of systemic inflammation and organ dysfunction, rather than relying solely on delayed laboratory values or qualitative image interpretation. By targeting clinically consequential endpoints, our framework extends beyond conventional severity classification toward outcome-oriented multimodal risk stratification. Early identification of high-risk patients has direct implications for triage, monitoring intensity, and timely initiation of evidence-based interventions. Prospective multicenter validation is required before this approach can be considered for clinical implementation or routine decision support.

## Supplementary Information

Below is the link to the electronic supplementary material.

Supplementary Material 1

## Data Availability

No datasets were generated or analysed during the current study.
