# Correction: Multimodal AI for early prediction of adverse clinical outcomes in acute pancreatitis

**DOI:** 10.1007/s00261-026-05657-0

**Published:** 2026-07-31

**Authors:** Ahmet Yasin Karkas, Yavuz B. Taktak, Burak Gultekin, Ziliang Hong, Halil Ertugrul Aktas, Deniz Seyithanoglu, Timurhan Cebeci, Alper Akin, Ece Elustu, Kerem Arisin, Ali Canturk, Mehmet Ilhan, Naci Senkal, Michael B. Wallace, Abraham F. Bezuidenhout, Frank H. Miller, Alpay Medetalibeyoglu, Mehmet Semih Cakir, Gorkem Durak, Ulas Bagci, Sukru Mehmet Erturk

**Affiliations:** 1 Department of Radiology, Istanbul University Faculty of Medicine, Istanbul, Turkey; 2 Department of Internal Medicine, Uskudar State Hospital, Istanbul, Turkey; 3 Department of Radiology, Northwestern University, Chicago, United States; 4 Department of Internal Medicine, Istanbul University Faculty of Medicine, Istanbul, Turkey; 5 Department of Public Health, Marmara University School of Medicine, Istanbul, Turkey; 6 Department of Radiology, Ministry of Health University Abdulhamid Han Training and Research Hospital, Istanbul, Turkey; 7 Department of General Surgery, Istanbul University Faculty of Medicine, Istanbul, Turkey; 8 Department of Medicine, Mayo Clinic, Jacksonville, United States; 9 Department of Radiology, Beth Israel Deaconess Medical Center, Boston, United States

**Correction to: Abdominal Radiology**

**https://doi.org/10.1007/s00261-026-05588-w**

In this article citation for the LIFEx software was missing and should have been ‘Nioche C, Orlhac F, Boughdad S, Reuzé S, Goya-Outi J, Robert C, Pellot-Barakat C, Soussan M, Frouin F, Buvat I. LIFEx: a freeware for radiomic feature calculation in multimodality imaging to accelerate advances in the characterization of tumor heterogeneity. Cancer Research. 2018;78(16):4786–4789’.

The original article has been corrected.

