# Supplementary material for: Multimodal AI for early prediction of adverse clinical outcomes in acute pancreatitis
Source: Abdom Radiol (NY). 2026 Jun 2;51(12):6430–42. doi: 10.1007/s00261-026-05588-w (PMC13768232; doi:10.1007/s00261-026-05588-w)
Supplement: Supplementary file 1 [file 261_2026_5588_MOESM1_ESM.docx]

**SUPPLEMENTARY**

**Fig. S1.** SHAP summary plots illustrating feature contributions to adverse outcome prediction in acute pancreatitis. Each dot represents an individual patient across five cross-validation folds. The x-axis shows SHAP values, indicating the direction and magnitude of each feature’s impact on the model output (positive values push the prediction toward an adverse outcome; negative values toward a favorable outcome). Features are ordered by overall importance (mean absolute SHAP value). Color represents the feature value, ranging from low (blue) to high (red). **(A)** Higher WBC, BUN/Cr, LDH, and CRP values, along with lower albumin levels, were associated with an increased risk of adverse outcomes. **(B)** Prominent contributing radiomic features included intensity-, shape-, and texture-based descriptors such as original_shape_MeshVolume, original_glrlm_RunLengthNonUniformity, wavelet-LLL_firstorder_Skewness, lbp-2D_firstorder_Mean, and wavelet-HLH_glszm_GrayLevelNonUniformity.

**CT Image Acquisition, Technical Details**

CECT scans were performed using the Canon TSX-305A/5K model, which features 320 detectors and 640 sections, and Brilliance-Philips tomography devices with 16 detectors. All patients were positioned supine with full inspiration. The imaging protocol consisted of a tube voltage of 120 kV, tube current modulation ranging from 50 to 150 mA, a pitch of 0.85–1, an image slice thickness of 1 mm, and a reconstruction interval of 1 mm. An iodinated contrast agent was administered via the cubital vein at a dose of 1.5 mL/kg, with a flow rate of 3 mL/s. Before contrast injection, kidney function was assessed in all patients, and informed consent was obtained after review of their medical history, including any allergies and chronic diseases. Portal venous phase images were acquired 65–70 s following the intravenous (IV) contrast injection.

**Supplemental Table S1.** Radiomic features and their potential biological interpretations in acute pancreatitis.

| **Region** | **Feature** | **Feature Category** | **Radiomic Interpretation** | **Possible Biological Interpretation** |
| --- | --- | --- | --- | --- |
| Pancreas | wavelet-LHL_firstorder_Minimum | Intensity | Lowest voxel intensity value within the wavelet-filtered image | May reflect attenuation heterogeneity related to edema, necrosis, reduced enhancement, or inflammatory tissue change. |
| Pancreas | wavelet-LLL_firstorder_Skewness | Intensity | Asymmetry of voxel intensity distribution in the low-frequency wavelet image | May reflect attenuation heterogeneity related to edema, necrosis, reduced enhancement, or inflammatory tissue change. |
| Pancreas | original_firstorder_Skewness | Intensity | Asymmetry of the original voxel intensity distribution | May reflect attenuation heterogeneity related to edema, necrosis, reduced enhancement, or inflammatory tissue change. |
| Pancreas | original_glrlm_RunLengthNonUniformity | Texture | Variability in the length of consecutive gray-level runs | May reflect heterogeneous pancreatic parenchymal architecture related to inflammatory edema, necrotic change, or evolving local tissue injury. |
| Pancreas | wavelet-HHL_gldm_HighGrayLevelEmphasis | Texture | Emphasis on higher gray-level intensities within local texture patterns | May reflect heterogeneous pancreatic parenchymal architecture related to inflammatory edema, necrotic change, or evolving local tissue injury. |
| Pancreas | wavelet-LHH_glszm_LargeAreaEmphasis | Texture | Presence of large homogeneous gray-level zones within the texture map | May reflect heterogeneous pancreatic parenchymal architecture related to inflammatory edema, necrotic change, or evolving local tissue injury. |
| Pancreas | wavelet-HHL_glcm_SumSquares | Texture | Measure of gray-level variance within local texture patterns | May reflect heterogeneous pancreatic parenchymal architecture related to inflammatory edema, necrotic change, or evolving local tissue injury. |
| Pancreas | original_shape_MeshVolume | Shape | Three-dimensional volumetric measurement of the segmented pancreas | May reflect pancreatic enlargement, swelling, or morphologic remodeling related to inflammation. |
| Peripancreatic edema | wavelet-HLH_glszm_GrayLevelNonUniformity | Texture | Variability of gray-level intensity values within edema texture zones | May reflect heterogeneous peripancreatic inflammatory extension, edema complexity, and variable surrounding tissue involvement. |
| Peripancreatic edema | lbp-2D_firstorder_Mean | Texture/ intensity | Mean intensity value derived from a local binary pattern-transformed texture image | May reflect heterogeneous peripancreatic inflammatory extension, edema complexity, and variable surrounding tissue involvement. |
| Peripancreatic edema | wavelet-LHH_glcm_ClusterShade | Texture | Measure of asymmetry within gray-level texture clusters | May reflect heterogeneous peripancreatic inflammatory extension, edema complexity, and variable surrounding tissue involvement. |
